# Supplementary material for: A Novel NKX2-5 Variant in a Child with Left Ventricular Noncompaction, Atrial Septal Defect, Atrioventricular Conduction Disorder, and Syncope
Source: J Clin Med. 2022 Jun 2;11(11):3171. doi: 10.3390/jcm11113171 (PMC9181799; doi:10.3390/jcm11113171)
Supplement: Supplementary file 1 [file jcm-11-03171-s001.zip › jcm-1732298-supplementary.pdf]

Supplementary Appendix

Methods

Mutation screening

After obtaining informed consent from a patient's parents, DNA was isolated from a sample of whole blood or heart tissue. NGS of 182 cardiac disorder-related genes associated with cardiomyopathies and channelopathies (Table S1) was performed using an Ion PGM System (Life Technologies, Carlsbad, CA, USA).

This custom panel utilized two separate PCR primer pools, yielding a total of 1,870 amplicons and used to generate target amplicon libraries. Genomic DNA samples were PCR-amplified using the custom panel and an Ion AmpliSeq Library Kit v2.0 (Life Technologies, Carlsbad, CA, USA). Individual samples were labeled using an Ion Xpress Barcode Adapters Kit (Life Technologies) and then pooled at equimolar concentrations. Emulsion PCR and ion sphere particle (ISP) enrichment were performed using the Ion PGM Hi-Q OT2 Kit (Life Technologies), according to the manufacturer's instructions. ISPs were loaded onto a 316 chip and sequenced using an Ion PGM Hi-Q Sequencing Kit (Life Technologies).

Sanger sequencing

For all candidate pathogenic variants that passed these selection criteria, Sanger sequencing was used to validate the NGS results. For this, the nucleotide sequences of amplified fragments were analyzed by direct sequencing in both directions by using the BigDye Terminator v3.1 Cycle Sequencing Kit (Applied Biosystems, Foster City, CA) and sequence analysis was performed using an ABI 3130xl automated sequencer (Applied Biosystems).

Data analysis and variant classification

Torrent Suite and Ion Reporter Software 5.0 (Life Technologies) were used to perform primary, secondary, and tertiary analyses, including optimized signal processing, base calling, sequence alignment, and variant analysis.

The allelic frequency of all detected variants was determined using the Exome Aggregation Consortium (ExAC) database and Human Genetic Variation Database (HGVD), which contain data of 1,208 Japanese individuals. All variants with a minor allelic frequency of ≥0.005 in the ExAC and HGVD populations were excluded. To evaluate the pathogenicity of the remaining variants, we utilized seven different *in silico* predictive algorithms: FATHMM, SIFT, PROVEAN, Align GVGD, MutationTaster2, PolyPhen-2, and CADD (Table S2). Variants predicted to be deleterious or pathogenic by at least five of the seven *in silico* algorithms were considered likely pathogenic.

Table S1. List of 182 analyzed genes of NGS.

Gene	Chromosome	NCBI Reference Sequence:	Sequence : (Start..End)	
ABCC9	12p12.1	NG_012819.1	NC_000012.11 (21950323..22094797, complement)	http://www.ncbi.nlm.nih.gov/gene/10060
ACAD9	3q21.3	NG_017064.1	NC_000003.12 (128879490..128913114)	https://www.ncbi.nlm.nih.gov/gene/28976
ACADVL	17p13.1	NG_007975.1	NC_000017.11 (7217125..7225267)	https://www.ncbi.nlm.nih.gov/gene/37
ACTC1	15q14	NG_007553.1	NC_000015.9 (35080297..35087927, complement)	http://www.ncbi.nlm.nih.gov/gene/70
ACTN2	1q42-q43	NG_009081.1	NC_000001.10 (236849754..236927931)	http://www.ncbi.nlm.nih.gov/gene/88
ADAMTS1	21q21.3	NC_000021.9	NC_000021.9 (26836287..26845409, complement)	https://www.ncbi.nlm.nih.gov/gene/9510
ADAMTS9	3p14.1	NC_000003.12	NC_000003.12 (64515654..64688000, complement)	https://www.ncbi.nlm.nih.gov/gene/56999
ADCK3	1q42.13	NG_012825.2	NC_000001.11 (226939339..226987545)	https://www.ncbi.nlm.nih.gov/gene/56997
AKAP9	7q21-q22	NG_011623.1	NC_000007.13 (91570181..91739987	http://www.ncbi.nlm.nih.gov/gene/10142
AMPD1	1p13.2	NG_008012.1	NC_000001.11 (114673098..114695618, complement)	https://www.ncbi.nlm.nih.gov/gene/270
ANK2	4q25-q27	NG_009006.2	NC_000004.11 (113739239..114304896)	http://www.ncbi.nlm.nih.gov/gene/287
ARFGEF2	20q13.13	NG_011490.1	NC_000020.11 (48921721..49036693)	https://www.ncbi.nlm.nih.gov/gene/10564
BAG3	10q25.2-q26.2	NG_016125.1	NC_000010.10 (121410859..121437331)	http://www.ncbi.nlm.nih.gov/gene/9531
BMP10	2p13.3	NG_032117.1	NC_000002.12 (68860916..68871517, complement)	https://www.ncbi.nlm.nih.gov/gene/27302
BMPR1A	10q22.3	NG_009362.1	NC_000010.10 (88516396..88684945)	http://www.ncbi.nlm.nih.gov/gene/657
BOLA3	2p13.1	NG_031910.1	NC_000002.12 (74135401..74147912, complement)	https://www.ncbi.nlm.nih.gov/gene/388962
BRAF	7q34	NG_007873.3	NC_000007.14 (140719327..140924928, complement)	https://www.ncbi.nlm.nih.gov/gene/673
C10orf2	10q24.31	NG_012624.1	NC_000010.11 (100987527..100994403)	https://www.ncbi.nlm.nih.gov/gene/56652
CACNA1C	12p13.3	NG_008801.2	NC_000012.11 (2079952..2807115)	http://www.ncbi.nlm.nih.gov/gene/775
CACNA2D1	7q21.11	NC_000007.14	NC_000007.14 (81946444..82443806, complement)	https://www.ncbi.nlm.nih.gov/gene/781
CACNB2	10p12	NG_016195.1	NC_000010.10 (18429373..18830688)	http://www.ncbi.nlm.nih.gov/gene/783
CALR3	19p13.11	NG_031959.2	NC_000019.9 (16589767..16607015, complement)	http://www.ncbi.nlm.nih.gov/gene/125972
CAPN3	15q15.1	NG_008660.1	NC_000015.9 (42646545..42704515)	http://www.ncbi.nlm.nih.gov/gene/825
CASQ2	1p13.1	NG_008802.1	NC_000001.11 (115700003..115768805, complement)	https://www.ncbi.nlm.nih.gov/gene/845
CASZ1	1p36.22	NC_000001.11	NC_000001.11 (10636604..10796676, complement)	https://www.ncbi.nlm.nih.gov/gene/54897

CAV3	3p25	NG_008797.2	NC_000003.11 (8775486..8788451)	http://www.ncbi.nlm.nih.gov/gene/859
CDKN1C	11p15.4	NG_008022.1	NC_000011.10 (2883218..2885804, complement)	https://www.ncbi.nlm.nih.gov/gene/1028
COL4A1	13q34	NG_011544.1	NC_000013.10 (110801310..110959496, complement)	http://www.ncbi.nlm.nih.gov/gene/1282
COL7A1	3p21.31	NG_007065.1	NC_000003.12 (48564073..48595302, complement)	https://www.ncbi.nlm.nih.gov/gene/1294
CPT2	1p32.3	NG_008035.1	NC_000001.11 (53196429..53214197)	https://www.ncbi.nlm.nih.gov/gene/1376
CSRP3	11p15.1	NG_011932.2	NC_000011.10 (19182030..19210571, complement)	https://www.ncbi.nlm.nih.gov/gene/8048
CTNNA3	10q21.3	NG_034072.1	NC_000010.11 (65912518..67696217, complement)	https://www.ncbi.nlm.nih.gov/gene/29119
DAAM1	14q23.1	NG_047127.1	NC_000014.9 (59188657..59371405)	https://www.ncbi.nlm.nih.gov/gene/23002
DAAM2	6p21.2	NC_000006.12	NC_000006.12 (39792366..39904877)	https://www.ncbi.nlm.nih.gov/gene/23500
DES	2q35	NG_008043.1	NC_000002.11 (220283099..220291461)	http://www.ncbi.nlm.nih.gov/gene/1674
DMD	Xp21.2	NG_012232.1	NC_000023.10 (31137345..33357726, complement)	http://www.ncbi.nlm.nih.gov/gene/1756
DMPK	19q13.32	NG_009784.1	NC_000019.10 (45769709..45782557, complement)	https://www.ncbi.nlm.nih.gov/gene/1760
DNAJC19	3q26.33	NG_022933.1	NC_000003.12 (180983709..180989774, complement)	https://www.ncbi.nlm.nih.gov/gene/131118
DSC2	18q12.1	NG_008208.1	NC_000018.9 (28645938..28682388, complement)	http://www.ncbi.nlm.nih.gov/gene/1824
DSG2	18q12.1	NG_007072.3	NC_000018.9 (29078027..29128814)	http://www.ncbi.nlm.nih.gov/gene/1829
DSP	6p24	NG_008803.1	NC_000006.11 (7541808..7586946)	http://www.ncbi.nlm.nih.gov/gene/1832
DTNA	18q12.1	NG_009201.1	NC_000018.10 (34493290..34891844)	https://www.ncbi.nlm.nih.gov/gene/1837
DVL1	1p36.33	NG_008048.1	NC_000001.11 (1335278..1349142, complement)	https://www.ncbi.nlm.nih.gov/gene/1855
EED	11q14.2	NG_029595.1	NC_000011.10 (86244384..86285420)	https://www.ncbi.nlm.nih.gov/gene/8726
ELN	7q11.23	NG_009261.1	NC_000007.13 (73442119..73484237)	http://www.ncbi.nlm.nih.gov/gene/2006
EMD	Xq28	NG_008677.1	NC_000023.10 (153607597..153609883)	http://www.ncbi.nlm.nih.gov/gene/2010
ERBB2	17q12	NG_007503.1	NC_000017.11 (39688084..39728662)	https://www.ncbi.nlm.nih.gov/gene/2064
ERBB4	2q34	NG_011805.1	NC_000002.12 (211375717..212538628, complement)	https://www.ncbi.nlm.nih.gov/gene/2066
EZH2	7q36.1	NG_032043.1	NC_000007.14 (148807372..148884349, complement)	https://www.ncbi.nlm.nih.gov/gene/2146
FBN2	5q23.3	NG_008750.1	NC_000005.10 (128257909..128538042, complement)	https://www.ncbi.nlm.nih.gov/gene/2201
FGF16	Xq21.1	NG_034050.1	NC_000023.11 (77447675..77456522)	https://www.ncbi.nlm.nih.gov/gene/8823
FGF9	13q12.11	NG_016272.1	NC_000013.11 (21671076..21704501)	https://www.ncbi.nlm.nih.gov/gene/2254

FGFR1	8p11.23	NG_007729.1	NC_000008.11 (38411138..38468834, complement)	https://www.ncbi.nlm.nih.gov/gene/2260
FGFR2	10q26.13	NG_012449.2	NC_000010.11 (121478330..121598458, complement)	https://www.ncbi.nlm.nih.gov/gene/2263
FKBP1A	20p13	NC_000020.11	NC_000020.11 (1368977..1393172, complement)	https://www.ncbi.nlm.nih.gov/gene/2280
FKBP1B	2p23.3	NC_000002.12	NC_000002.12 (24033205..24067743)	https://www.ncbi.nlm.nih.gov/gene/2281
FKTN	9q31.2	NG_008754.1	NC_000009.12 (105558117..105655950)	https://www.ncbi.nlm.nih.gov/gene/2218
FLNA	Xq28	NG_011506.1	NC_000023.11 (154348532..154374638, complement)	https://www.ncbi.nlm.nih.gov/gene/2316
FXN	9q21.11	NG_008845.2	NC_000009.12 (69035563..69079077)	https://www.ncbi.nlm.nih.gov/gene/2395
GAA	17q25.2-q25.3	NG_009822.1	NC_000017.10 (78075339..78093680)	http://www.ncbi.nlm.nih.gov/gene/2548
GATA4	8p23.1-p22	NG_008177.1	NC_000008.10 (11534433..11617510)	http://www.ncbi.nlm.nih.gov/gene/2626
GBE1	3p12.2	NG_011810.1	NC_000003.12 (81489699..81761799, complement)	https://www.ncbi.nlm.nih.gov/gene/2632
GFRA1	10q25.3	NG_050620.1	NC_000010.11 (116056925..116273645, complement)	https://www.ncbi.nlm.nih.gov/gene/2674
GFRA2	8p21.3	NG_029215.1	NC_000008.11 (21690403..21789296, complement)	https://www.ncbi.nlm.nih.gov/gene/2675
GLA	Xq22	NG_007119.1	NC_000023.10 (100652779..100663001, complement)	http://www.ncbi.nlm.nih.gov/gene/2717
GPD1L	3p22.3	NG_023375.1	NC_000003.11 (32148003..32210207)	http://www.ncbi.nlm.nih.gov/gene/23171
HADHA	2p23.3	NG_007121.1	NC_000002.12 (26190635..26244726, complement)	https://www.ncbi.nlm.nih.gov/gene/3030
HAS2	8q24.13	NC_000008.11	NC_000008.11 (121613031..121641390, complement)	https://www.ncbi.nlm.nih.gov/gene/3037
HBB	11p15.4	NG_059281.1	NC_000011.10 (5225466..5227071, complement)	https://www.ncbi.nlm.nih.gov/gene/3043
HCCS	Xp22.2	NG_016460.1	NC_000023.11 (11111286..11123086)	https://www.ncbi.nlm.nih.gov/gene/3052
HCN4	15q24.1	NG_009063.1	NC_000015.9 (73612200..73661605, complement)	http://www.ncbi.nlm.nih.gov/gene/10021
HEY2	6q22.31	NC_000006.12	NC_000006.12 (125747639..125762243)	https://www.ncbi.nlm.nih.gov/gene/23493
HMGCL	1p36.11	NG_013061.1	NC_000001.11 (23801877..23825459, complement)	https://www.ncbi.nlm.nih.gov/gene/3155
ITGA7	12q13.2	NG_012343.1	NC_000012.12 (55684568..55716037, complement)	https://www.ncbi.nlm.nih.gov/gene/3679
JARID2	6p22.3	NC_000006.12	NC_000006.12 (15245975..15522042)	https://www.ncbi.nlm.nih.gov/gene/3720
JUP	17q21	NG_009090.2	NC_000017.10 (39910859..39942964, complement)	http://www.ncbi.nlm.nih.gov/gene/3728
KCNE1	21q22.12	NG_009091.1	NC_000021.8 (35790910..35884573, complement)	http://www.ncbi.nlm.nih.gov/gene/3753
KCNE2	21q22.12	NG_008804.1	NC_000021.8 (35736323..35743440)	http://www.ncbi.nlm.nih.gov/gene/9992
KCNE3	11q13.4	NG_011833.1	NC_000011.9 (74165886..74178600, complement)	http://www.ncbi.nlm.nih.gov/gene/10008

KCNH2	7q36.1	NG_008916.1	NC_000007.13 (150642044..150675402, complement)	http://www.ncbi.nlm.nih.gov/gene/3757
KCNJ2	17q24.3	NG_008798.1	NC_000017.10 (68164757..68176189)	http://www.ncbi.nlm.nih.gov/gene/3759
KCNQ1	11p15.5	NG_008935.1	NC_000011.9 (2466221..2870340)	http://www.ncbi.nlm.nih.gov/gene/3784
KRAS	12p12.1	NG_007524.1	NC_000012.11 (25358180..25403870, complement)	http://www.ncbi.nlm.nih.gov/gene/3845
LAMP2	Xq24	NG_007995.1	NC_000023.10 (119560003..119603204, complement)	http://www.ncbi.nlm.nih.gov/gene/3920
LDB3	10q22.3-q23.2	NG_008876.1	NC_000010.10 (88426542..88495829)	http://www.ncbi.nlm.nih.gov/gene/11155
LMNA	1q22	NG_008692.2	NC_000001.10 (156052369..156109880)	http://www.ncbi.nlm.nih.gov/gene/4000
LMX1B	9q33.3	NG_017039.1	NC_000009.12 (126614443..126701032)	https://www.ncbi.nlm.nih.gov/gene/4010
LRPPRC	2p21	NG_008247.1	NC_000002.12 (43886224..43996005, complement)	https://www.ncbi.nlm.nih.gov/gene/10128
MADD	11p11.2	NG_029462.1	NC_000011.10 (47269376..47330031)	https://www.ncbi.nlm.nih.gov/gene/8567
MBL2	10q21.1	NG_008196.1	NC_000010.11 (52764977..52772847, complement)	https://www.ncbi.nlm.nih.gov/gene/4153
MED1	17q12	NG_046996.1	NC_000017.11 (39404285..39451281, complement)	https://www.ncbi.nlm.nih.gov/gene/5469
MEST	7q32.2	NG_009226.1	NC_000007.14 (130486175..130506297)	https://www.ncbi.nlm.nih.gov/gene/4232
MIB1	18q11.2	NG_033272.2	NC_000018.10 (21740793..21870957)	https://www.ncbi.nlm.nih.gov/gene/57534
MIPEP	13q12.12	NG_052977.1	NC_000013.11 (23730189..23889448, complement)	https://www.ncbi.nlm.nih.gov/gene/4285
MLYCD	16q23.3	NG_009079.1	NC_000016.10 (83899125..83916182)	https://www.ncbi.nlm.nih.gov/gene/23417
MMACHC	1p34.1	NG_013378.1	NC_000001.11 (45500184..45511266)	https://www.ncbi.nlm.nih.gov/gene/25974
MRPS22	3q23	NG_012174.1	NC_000003.12 (139344014..139357129)	https://www.ncbi.nlm.nih.gov/gene/56945
MTO1	6q13	NG_032856.1	NC_000006.12 (73461731..73501456)	https://www.ncbi.nlm.nih.gov/gene/25821
MYBPC3	11p11.2	NG_007667.1	NC_000011.9 (47352957..47374253, complement)	http://www.ncbi.nlm.nih.gov/gene/4607
MYCN	2p24.3	NG_007457.1	NC_000002.12 (15940438..15947007)	https://www.ncbi.nlm.nih.gov/gene/4613
MYH11	16p13.11	NG_009299.1	NC_000016.9 (15796992..15950887, complement)	http://www.ncbi.nlm.nih.gov/gene/4629
MYH6	14q12	NG_023444.1	NC_000014.8 (23849942..23878836, complement)	http://www.ncbi.nlm.nih.gov/gene/4624
MYH7	14q12	NG_007884.1	NC_000014.8 (23881947..23904870, complement)	http://www.ncbi.nlm.nih.gov/gene/4625
MYH7B	20q11.22	NG_016984.2	NC_000020.11 (34955835..35002437)	https://www.ncbi.nlm.nih.gov/gene/57644
MYL2	12q24.11	NG_007554.1	NC_000012.11 (111348623..111358404, complement)	http://www.ncbi.nlm.nih.gov/gene/4633
MYL3	3p21.3-p21.2	NG_007555.2	NC_000003.11 (46899357..46904973, complement)	http://www.ncbi.nlm.nih.gov/gene/4634
MYLK	3q21	NG_029111.1	NC_000003.11 (123331143..123603149, complement)	http://www.ncbi.nlm.nih.gov/gene/4638

MYOZ2	4q26-q27	NG_029747.1	NC_000004.11 (120056939..120108944)	http://www.ncbi.nlm.nih.gov/gene/51778
NEXN	1p31.1	NG_016625.1	NC_000001.11 (77888515..77948643)	https://www.ncbi.nlm.nih.gov/gene/91624
NFATC1	18q23	NG_029226.1	NC_000018.10 (79395772..79529323)	https://www.ncbi.nlm.nih.gov/gene/4772
NKX2-5	5q34	NG_013340.1	NC_000005.9 (172659107..172662315, complement)	http://www.ncbi.nlm.nih.gov/gene/1482
NNT	5p12	NG_032869.1	NC_000005.10 (43601092..43705566)	https://www.ncbi.nlm.nih.gov/gene/23530
NR0B1	Xp21.2	NG_009814.1	NC_000023.11 (30304422..30309378, complement)	https://www.ncbi.nlm.nih.gov/gene/190
NRAS	1p13.2	NG_007572.1	NC_000001.10 (115247085..115259515, complement)	http://www.ncbi.nlm.nih.gov/gene/4893
NRG1	8p12	NG_012005.2	NC_000008.11 (31639222..32771716)	https://www.ncbi.nlm.nih.gov/gene/3084
NSD1	5q35.3	NG_009821.1	NC_000005.10 (177131835..177300213)	https://www.ncbi.nlm.nih.gov/gene/64324
NUMB	14q24.2-q24.3	NG_029061.2	NC_000014.9 (73275210..73458580, complement)	https://www.ncbi.nlm.nih.gov/gene/8650
NUMBL	19q13.2	NC_000019.10	NC_000019.10 (40665905..40690658, complement)	https://www.ncbi.nlm.nih.gov/gene/9253
PKP2	12p11	NG_009000.1	NC_000012.11 (32943680..33049780, complement)	http://www.ncbi.nlm.nih.gov/gene/5318
PLEC	8q24.3	NG_012492.1	NC_000008.11 (143915147..143976800, complement)	https://www.ncbi.nlm.nih.gov/gene/5339
PLEKHM2	1p36.21	NG_053033.1	NC_000001.11 (15681506..15734769)	https://www.ncbi.nlm.nih.gov/gene/23207
PLN	6q22.1	NG_009082.1	NC_000006.11 (118869442..118881587)	http://www.ncbi.nlm.nih.gov/gene/5350
PMP22	17p12	NG_007949.1	NC_000017.11 (15229777..15265373, complement)	https://www.ncbi.nlm.nih.gov/gene/5376
POLG	15q26.1	NG_008218.2	NC_000015.10 (89316305..89334795, complement)	https://www.ncbi.nlm.nih.gov/gene/5428
PRDM16	1p36.32	NG_029576.1	NC_000001.11 (3068227..3438621)	https://www.ncbi.nlm.nih.gov/gene/63976
PRKAG2	7q36.1	NG_007486.1	NC_000007.13 (151253200..151574316, complement)	http://www.ncbi.nlm.nih.gov/gene/51422
PTGS2	1q31.1	NG_028206.2	NC_000001.11 (186671812..186680427, complement)	https://www.ncbi.nlm.nih.gov/gene/5743
PTK2	8q24.3	NG_029467.1	NC_000008.11 (140658382..141002079, complement)	https://www.ncbi.nlm.nih.gov/gene/5747
PTPN11	12q24	NG_007459.1	NC_000012.11 (112856536..112947717)	http://www.ncbi.nlm.nih.gov/gene/5781
RAD54L2	3p21.2	NC_000003.12	NC_000003.12 (51538683..51668660)	https://www.ncbi.nlm.nih.gov/gene/23132
RAF1	3p25	NG_007467.1	NC_000003.11 (12625100..12705700, complement)	http://www.ncbi.nlm.nih.gov/gene/5894
RANGRF	17p13.1	NG_028189.1	NC_000017.11 (8288497..8290092)	https://www.ncbi.nlm.nih.gov/gene/29098
RBM20	10q25.2	NG_021177.1	NC_000010.11 (110641933..110839471)	https://www.ncbi.nlm.nih.gov/gene/282996

RIT1	1q22	NG_033885.1	NC_000001.11 (155897808..155911402, complement)	https://www.ncbi.nlm.nih.gov/gene/6016
RPS6KA3	Xp22.12	NG_007488.1	NC_000023.11 (20149911..20267514, complement)	https://www.ncbi.nlm.nih.gov/gene/6197
RPS7	2p25	NG_011744.1	NC_000002.11 (3622853..3628509)	http://www.ncbi.nlm.nih.gov/gene/6201
RYR1	19q13.2	NG_008866.1	NC_000019.10 (38433700..38587564)	https://www.ncbi.nlm.nih.gov/gene/6261
RYR2	1q43	NG_008799.2	NC_000001.10 (237205510..237997288)	http://www.ncbi.nlm.nih.gov/gene/6262
SCN1B	9q13.1	NG_013359.1	NC_000019.9 (35521555..35531353)	http://www.ncbi.nlm.nih.gov/gene/6324
SCN3B	11q23.3	NG_016283.1	NC_000011.9 (123499895..123525315, complement)	http://www.ncbi.nlm.nih.gov/gene/55800
SCN4B	11q23.3	NG_011710.1	NC_000011.9 (118004092..118023630, complement)	http://www.ncbi.nlm.nih.gov/gene/6330
SCN5A	3p21	NG_008934.1	NC_000003.11 (38589553..38691164, complement)	http://www.ncbi.nlm.nih.gov/gene/6331
SCO2	22q13.33	NG_016235.1	NC_000022.11 (50523568..50526439, complement)	https://www.ncbi.nlm.nih.gov/gene/9997
SCRIB	8q24.3	NG_030583.1	NC_000008.11 (143790920..143815379, complement)	https://www.ncbi.nlm.nih.gov/gene/23513
SDHA	5p15.33	NG_012339.1	NC_000005.10 (218223..264816)	https://www.ncbi.nlm.nih.gov/gene/6389
SDHAF1	19q13.12	NG_016869.1	NC_000019.10 (35995188..35996318)	https://www.ncbi.nlm.nih.gov/gene/644096
SDHB	1p36.13	NG_012340.1	NC_000001.11 (17018722..17054170, complement)	https://www.ncbi.nlm.nih.gov/gene/6390
SDS	12q24.13	NC_000012.12	NC_000012.12 (113392445..113403887, complement)	https://www.ncbi.nlm.nih.gov/gene/10993
SGCD	5q33-q34	NG_008693.2	NC_000005.9 (155462147..156194799)	http://www.ncbi.nlm.nih.gov/gene/6444
SLC22A5	5q31.1	NG_008982.2	NC_000005.10 (132369704..132395614)	https://www.ncbi.nlm.nih.gov/gene/6584
SLC25A20	3p21.31	NG_008171.1	NC_000003.12 (48856923..48898993, complement)	https://www.ncbi.nlm.nih.gov/gene/788
SLC25A4	4q35	NG_013001.1	NC_000004.11 (186064417..186071538)	http://www.ncbi.nlm.nih.gov/gene/291
SLC25A4	4q35.1	NG_013001.1	NC_000004.12 (185143263..185150384)	https://www.ncbi.nlm.nih.gov/gene/291
SLC25A5	Xq24	NG_013262.1	NC_000023.11 (119468400..119471396)	https://www.ncbi.nlm.nih.gov/gene/292
SLC52A2	8q24.3	NG_032872.1	NC_000008.11 (144358547..144361286)	https://www.ncbi.nlm.nih.gov/gene/79581
SMAD3	15q22.33	NG_011990.1	NC_000015.9 (67358036..67487533)	http://www.ncbi.nlm.nih.gov/gene/4088
SMAD7	18q21.1	NG_023330.1	NC_000018.10 (48919853..48950711, complement)	https://www.ncbi.nlm.nih.gov/gene/4092
SMARCA4	19p13.2	NG_011556.2	NC_000019.10 (10960922..11062282)	https://www.ncbi.nlm.nih.gov/gene/6597
SNTA1	20q11.2	NG_011622.1	NC_000020.10 (31995763..32031698, complement)	http://www.ncbi.nlm.nih.gov/gene/6640
SOS1	2p21	NG_007530.1	NC_000002.11 (39208690..39347686, complement)	http://www.ncbi.nlm.nih.gov/gene/6654
SQSTM1	5q35.3	NG_011342.1	NC_000005.10 (179806388..179838078)	https://www.ncbi.nlm.nih.gov/gene/8878
STARD3	17q11-q12		NC_000017.10 (37793333..37820454)	http://www.ncbi.nlm.nih.gov/gene/10948

TAZ	Xq28	NG_009634.1	NC_000023.10 (153639877..153650065)	http://www.ncbi.nlm.nih.gov/gene/6901
TBX20	7p14.2	NG_015805.1	NC_000007.14 (35199936..35254100, complement)	https://www.ncbi.nlm.nih.gov/gene/57057
TBX5	12q24.1	NG_007373.1	NC_000012.11 (114791735..114846247, complement)	http://www.ncbi.nlm.nih.gov/gene/6910
TCAP	17q12	NG_008892.1	NC_000017.11 (39665346..39666554)	https://www.ncbi.nlm.nih.gov/gene/8557
TGFB3	14q24.3	NG_011715.1	NC_000014.9 (75958061..75983011, complement)	https://www.ncbi.nlm.nih.gov/gene/7043
TGFBR1	9q22	NG_007461.1	NC_000009.11 (101867412..101916474)	http://www.ncbi.nlm.nih.gov/gene/7046
TGFBR2	3p22	NG_007490.1	NC_000003.11 (30647994..30735634)	http://www.ncbi.nlm.nih.gov/gene/7048
TMEM43	3p25.1	NG_008975.1	NC_000003.11 (14166440..14185180)	http://www.ncbi.nlm.nih.gov/gene/79188
TMEM70	8q21.11	NG_016618.1	NC_000008.11 (73976142..73982783)	https://www.ncbi.nlm.nih.gov/gene/54968
TNNC1	3p21.1	NG_008963.1	NC_000003.11 (52485107..52488057, complement)	http://www.ncbi.nlm.nih.gov/gene/7134
TNNI3	19q13.4	NG_007866.2	NC_000019.9 (55663135..55669100, complement)	http://www.ncbi.nlm.nih.gov/gene/7137
TNNT2	1q32	NG_007556.1	NC_000001.10 (201328136..201346836, complement)	http://www.ncbi.nlm.nih.gov/gene/7139
TPM1	15q22.1	NG_007557.1	NC_000015.9 (63334838..63364114)	http://www.ncbi.nlm.nih.gov/gene/7168
TTN	2q31.2	NG_011618.3	NC_000002.12 (178525989..178807423, complement)	https://www.ncbi.nlm.nih.gov/gene/7273
TTR	18q12.1	NG_009490.1	NC_000018.10 (31591767..31599024)	https://www.ncbi.nlm.nih.gov/gene/7276
VANGL2	1q23.2	NG_023420.1	NC_000001.11 (160400574..160428678)	https://www.ncbi.nlm.nih.gov/gene/57216
VCL	10q22.2	NG_008868.1	NC_000010.10 (75757836..75879918)	http://www.ncbi.nlm.nih.gov/gene/7414
YAP1	11q22.1	NG_029530.1	NC_000011.10 (102109957..102233423)	https://www.ncbi.nlm.nih.gov/gene/10413
YWHAE	17p13.3	NG_009233.1	NC_000017.11 (1344539..1400262, complement)	https://www.ncbi.nlm.nih.gov/gene/7531
ZNF25	10p11.21	NC_000010.11	NC_000010.11 (37949572..37976655, complement)	https://www.ncbi.nlm.nih.gov/gene/219749

Table S2. Silico predictive algorithms used in the study.

Category	Basis	Name	Website	Prediction Threshold
Missense prediction	Evolutionary conservation	SIFT	http://sift.jcvi.org	<0.05 Deleterious<br>>0.05 Tolerated
Missense prediction	Protein structure/function and evolutionary conservation	Align GVGD	http://agvgd.iarc.fr/agvgd_input.php	≧C15 Probably Damaging
		Mutation Taster	http://www.mutationtaster.org	Disease causing
		Polyphen-2	http://genetics.bwh.harvard.edu/pph2	0.85 to 1 Probably Damage<br>0.15 to 0.85 Possibly Damage
Missense and insertion/deletions prediction	Contrasts annotations of fixed/nearly fixed derived alleles in humans with simulated variants	CADD	http://cadd.gs.washington.edu	≧20 1% most deleterious<br>≧30 0.1%most deleterious

Reference

1.Richards S, Aziz N, Bale S, Bick D, Das S, Gastier-Foster J, Grody WW, Hegde M, Lyon E, Spector E, Voelkerding K, Rehm HL. Standards and guidelines for the interpretation of sequence variants: a joint consensus recommendation of the American College of Medical Genetics and Genomics and the association for molecular autopsy. Genet Med 2015;17:405–423.

Table S3. Variant identified in the patient

Gene	Protein	NM_004387.1	dbSNP	gnomAD	HGVD	SIFT	polyphen2	GVGD	Mutation Taster	CADD	Clin Var	ACMG classification
NKX2-5	p.Phe86fs	c.255_256delCT	n/a	n/a	n/a	n/a	n/a	n/a	n/a	28.7	n/a	PM2
